# Awe Guards My Creativity: The Interactive Effect of Perceived Abusive Supervisory Behavior, Dispositional Awe, and Creative Self-Efficacy on Chinese Employee Creativity

**DOI:** 10.3389/fsoc.2020.00051

**Published:** 2020-07-14

**Authors:** Cynthia Atamba, Anastasia Popelnukha, Farida Lukoko Ibrahim

**Affiliations:** School of Management, Department of Business Administration, University of Science and Technology of China, Hefei, China

**Keywords:** awe, abusive supervision, self-efficacy, creativity, emotions as social information

## Abstract

The study examined the responses of employees to supervisors who exhibited abusive behavior and invoked dispositional awe to influence their followers. The proposition is that two divergent predictors of supervisor effectiveness interact to affect the behavior of subordinates. The purpose of this study was to examine the interactive effect of perceived abusive supervisory behavior and perceived supervisor dispositional awe on employee creative self-efficacy and creativity. To test the proposed model, we collected cross-sectional data from 196 working professionals pursuing their Masters of Business Administration (MBA) at a large university in China. Our findings confirmed that perceived abusive supervisory behavior and perceived supervisor dispositional awe were predictors of employee creativity. Also, perceived supervisor dispositional awe moderated the relationship between perceived abusive supervisory behavior and employee creative self-efficacy. The theoretical and practical implications for leaders and organizations were discussed.

## Introduction

Research indicates that ~50% of employees in the United States of America consider their supervisors to be abusive (Namie and Namie, 2000; Tepper et al., 2011). Abusive supervisory behavior is conceptualized as verbal and non-verbal hostile behaviors that supervisors exhibit to employees (Tepper, 2000). This hostility includes silent treatment, ridiculing subordinates in public, outwardly expressing anger, or being rude. Due to the intensity of abusive supervisory behavior, scholars have found that it negatively affects employee creativity. However, a handful of studies exploring the link between perceived abusive supervisory behavior and employee creativity have produced inconsistent findings. Some studies tentatively suggest a curvilinear relationship (Lee et al., 2013), while others demonstrate a negative relationship (Rauniyar et al., 2017; Zheng and Liu, 2017). Such contradictory empirical evidence indicates that fundamental questions remain unanswered. For instance, research has not examined why and how perceived abusive supervisory behavior influences creativity.

The current study aims to answer these fundamental questions while using emotions as social information (EASI; Van Kleef, 2016, 2017) model as the theoretical backdrop. The EASI model indicates that inconsistent supervisor behaviors and emotional expressions affect employee work-related perceptions through altered social cognition and emotions (Van Kleef, 2017). Therefore, we argue that employees may consider abusive supervision as a negative emotional stressor and as a reflection of their work-related abilities such as their creativity. Using a multi-component approach, we conceptualized creativity as the employee's ability and willingness to engage in various processes of creating paradigm shifts by challenging the existing knowledge and understanding (Amabile, 1983; Sternberg, 1985; Simonton, 1997). In this work, we focused on the cognitive mechanism of the EASI model as previous studies demonstrated that affective reactions might converge with cognitions (Wang et al., 2017). The EASI model also posits that the expresser's characteristics may change how observers interpret and react to other's actions and emotions (Van Kleef, 2017; Deng et al., 2020). Drawing on this idea, we argue that supervisor dispositional awe predicts an employee's evaluation of their creative self-efficacy and creativity. Shiota et al. (2006) conceptualized dispositional awe as the individual differences in the perception of awe-related experiences. Several studies have revealed that awe elicited by leaders promotes creativity (e.g., Duffour et al., 2017). In an organizational context, dispositional awe can be elicited by supervisors who are experts in their field and whose work is of the highest quality (Pastor et al., 2002; Keltner and Haidt, 2003; Gordon et al., 2017). Supervisors high in dispositional awe possess high intellectual character strengths related to creativity (Güsewell and Ruch, 2012), higher tolerance to uncertainty (Li et al., 2019), and persuasion (Griskevicius et al., 2010). These characteristics in supervisors are likely to promote employee openness to new ideas (Shiota et al., 2006) and promote convergent creativity (Isen et al., 1987; De Dreu and Van Lange, 1995; Baas et al., 2008). In sum, supervisor dispositional awe influences employees' creative self-efficacy and creativity.

Research has shown that leadership is crucial for enhancing organization-based outcomes (Ford, 1996; Zhang et al., 2015; Li C. R. et al., 2017) such as employee performance. Previous research explored how abusive (Liu et al., 2016), transformational (Li et al., 2015; Tung, 2016), visionary (Zhou et al., 2018), servant (Yang et al., 2017), and authoritarian (Gu et al., 2018) leaders affected employee creativity in China. The increased interest in leadership behavior (e.g., Liu et al., 2012) emphasizes the need to further our understanding of why and how the behavior of managers such as abusive supervisors affects employees. The detrimental effects of abusive supervisors on employee creativity has been thoroughly explored (e.g., Jiang and Tang, 2016; Hussain and Sia, 2017). However, recent findings by Fiset et al. (2019) have challenged the notion that inconsistent leadership behavior can not coincide with influencing employee perceptions. Their results showed that leaders could simultaneously demonstrate both abusive supervisory behavior and leadership vision to influence task performance (Fiset et al., 2019). The objective of this study was to explore other conflicting leader behaviors that influence employee creative self-efficacy and employee creativity.

**Figure 1:**
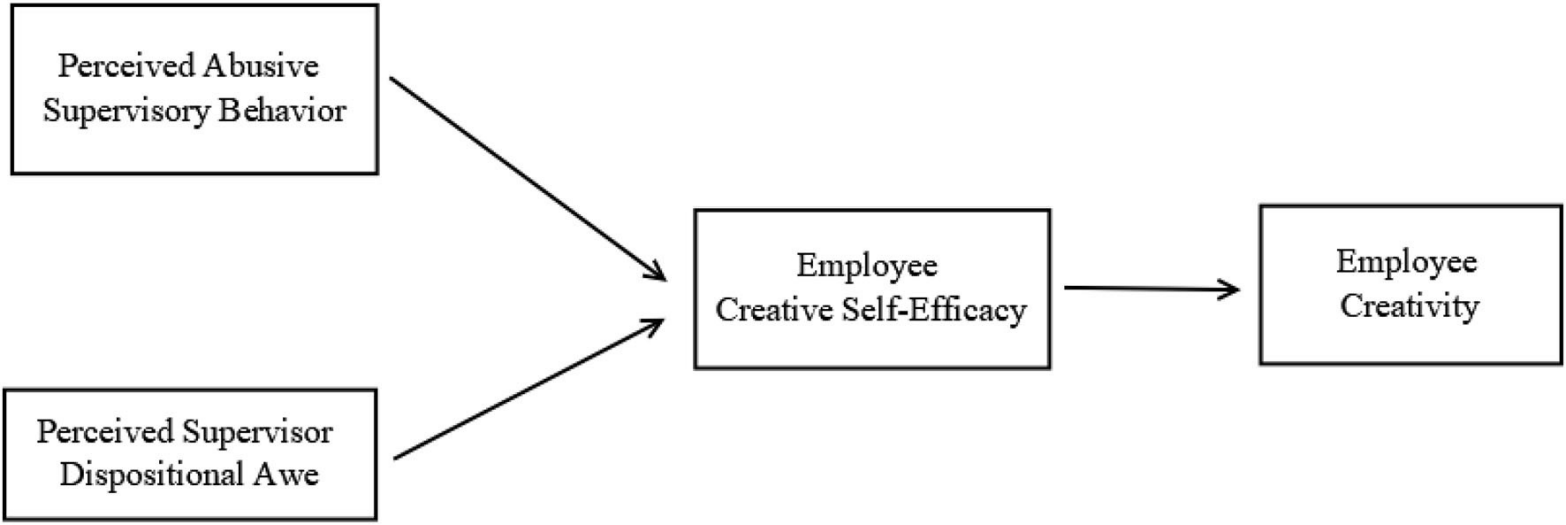
Proposed study model.

The current study offers two contributions to literature. First, the current study offers alternative theoretical explanations that may allow perceived abusive supervisory behavior to influence employees' attitudes and performance positively as shown in the proposed study model outlined in Figure 1. It examined the interactive effects of perceived supervisory abuse and dispositional awe on employee creativity (Namie and Namie, 2000; Tepper et al., 2011; Vogel and Mitchell, 2017) and employee well-being (Oh and Farh, 2017; Vogel and Mitchell, 2017). Previous research has separately examined supervisors who were often celebrated in corporate circles, and those whose behaviors were considered abusive (Oh and Farh, 2017). However, there have been instances when the same supervisor has been considered as visionary but later regarded as abusive (e.g., Steve Jobs; Isaacson, 2012). To reconcile these inconsistent perceptions of the same supervisor, emotion as social information model (Van Kleef et al., 2009; Van Kleef, 2017) was used to explain how perceived abusive supervisory behavior and perceived supervisor dispositional awe predict employee creative self-efficacy. The EASI model (Van Kleef et al., 2009; Van Kleef, 2017) has been previously used to explain how emotional expressions can influence the target's attributions (Hillebrandt and Barclay, 2017) and motivation (Wang et al., 2017).

Second, the current study contributes to the literature on positive psychology by exploring dispositional awe as a predictor and moderator (Gordon et al., 2017; Hentrup et al., 2019). Previous research focused on factors that enhance employee creative self-efficacy and creativity, such as promotions (Yunshu et al., 2019) and cross-cultural influences (Farmer et al., 2003). The most consistent and stable predictors have been trait- and state-based emotions such as awe (Li M. et al., 2017). To explore the influences of specific emotion upon social cognition (Shiota et al., 2006), we tested the hypothesis that dispositional awe boosts employee creative self-efficacy and promotes employee creativity. To the best of our knowledge, this is the first study to examine supervisor dispositional awe as a predictor of employee creative self-efficacy.

The study was divided into seven sections. First, we discussed perceived abusive supervisory behavior and perceived supervisor dispositional awe as predictors of employee creative self-efficacy in China. This part is followed by the hypothesis development for the moderating mechanisms, moderation-mediated model, research design, results, and discussion.

## Theoretical Background and Hypotheses

### Employee Creativity in China

Researchers often use the multi-factorial approach to measure creativity. Creativity is defined as a process that involves refining the current understanding of a phenomenon and then generating new concepts and ideas (Amabile, 1983; Sternberg, 1985; Simonton, 1997). Creativity is crucial for corporate survival (Amabile, 2017) as it enables organizations to produce exceptional products that create a competitive advantage (Shalley and Gilson, 2004). According to Tang et al. (2017), creativity must be recognized and studied within the cultural and social systems that it has originated. To emphasize these assertions, researchers have reported a significant relationship between cultural variables and creativity in Turkey (Gumusluoglu and Ilsev, 2009), Korea (Shin and Zhou, 2003), and China (Bai et al., 2017). These studies are in line with the assertion that collectivist cultures such as China aspire to transition from being manufacturers to innovators. The cultural norms and values in China affect leadership (Casimir and Waldman, 2007), and creativity (Zhang et al., 2015). Specifically, modernity, traditionality (Leong and Chang, 2003; Farh et al., 2007), and *guanxi* (Zhang et al., 2015) have been identified as essential cultural norms and values that explain creativity in China. While most studies on creativity have focused on theories and operations of organizations in Western cultures, insights into the dynamics in China have increased researcher interest (Jackson et al., 2014). Hence, this research intends to examine how and when leadership characteristics may influence employee creativity in emerging economies such as China.

### Employee Creative Self-Efficacy Mediates the Relationship Between Perceived Supervisor Abuse and Employee Creativity

Tierney and Farmer (2002) elaborated on Bandura's (1986) theory of self-efficacy by emphasizing that employee creative self-efficacy was the dominant precursor of employee creativity. In this study, creative self-efficacy was characterized by a secure connection with general creative functioning rather than creativity limited to specific fields (Kaufman et al., 2016). Creative self-efficacy, defined as “the belief (that) one can produce creative outcomes” (Tierney and Farmer, 2002, p. 1138), can be shaped by internal factors such as personal experience, physiological or emotional states, and personality, and by external factors including culture and socioeconomic status (Beghetto and Plucker, 2006). Given the vital role that leadership plays in shaping one's self-concept (Zheng and Liu, 2017), the current study builds on previous research to investigate the effect of abusive supervisory behavior on employee creative self-efficacy.

Researchers have highlighted trust, confidence, and praise as the critical components of employee creative self-efficacy (Tierney and Farmer, 2002; Farmer and Tierney, 2017). Abusive supervisory behavior negatively influences employee creative self-efficacy in three areas: social persuasion, task mastery, and physiological well-being (Jiang et al., 2019). First, employees rely on their supervisors and co-workers for social persuasion. Social persuasion is defined as the verbal encouragement that helps an employee to believe that they can accomplish creative tasks (Daniels, 2008). Social persuasion serves as verbal feedback (Bandura, 1986) that boosts supervisor-subordinate trust, inspires confidence, and rewards an employee through praise (Tierney and Farmer, 2002; Farmer and Tierney, 2017). Abusive supervisory behavior, such as silent treatment and public embarrassment (Tepper, 2000), convinces the employees that they are poor performers, which influences their creative self-efficacy. Second, employees enjoy feelings of achievement when they complete tasks that require a lot of time and effort (Locke et al., 1984). A good supervisor will publicly acknowledge and applaud these accomplishments, but an abusive one only focuses on the little failures and mistakes (Tepper, 2000). When supervisors fail to give credit for tasks requiring effort, it makes employees doubt their competence and ability to fulfill creative tasks. Third, abusive supervisory behavior induces strong emotions such as anger, fear, and sadness (Oh and Farh, 2017), which can be detrimental to psychological well-being and inhibit the development of creative self-efficacy (Tierney and Farmer, 2002). In sum, employees experiencing abusive supervision have low creative self-efficacy and low creativity. We, therefore, posit that:

***Hypothesis 1****: Employee creative self-efficacy mediates the relationship between perceived abusive supervisory behavior and employee creativity*.

### Employee Creative Self-Efficacy Mediates the Relationship Between Perceived Supervisor Dispositional Awe and Employee Creativity

Trait-based positive emotions, such as awe, have been shown to influence employee creativity (Guan et al., 2018). Since Shiota et al.'s (2006) conceptualization of dispositional awe, researchers have explored its effect on individual self-concepts. Previous research explored the effect of dispositional awe and happiness on individual self-concepts (Shiota et al., 2006). However, to the best of our knowledge, no studies have examined the relationship between supervisor dispositional awe and employee creative self-efficacy.

The appraisal tendency framework (Keltner et al., 2003; Lerner et al., 2015) states that dispositional awe influences emotional responses and guides subsequent cognitive processes in ways that are consistent with the emotion-specific stimuli. Guided by this framework, Keltner and Haidt (2003) outlined two features that are central to the feeling of awe: perceived vastness and the need for accommodation. Perceived vastness refers to experiences that are felt to be larger than life. In the case of individuals, awe can arise from their physical size, social status, fame, or authority. The need for accommodation refers to the reorganization of previous beliefs to understand the new stimulus. These two features prompt individuals to explore the environment, seek new information, and find new ways of resolving problems (Shiota et al., 2006). Dispositional awe has been linked to extraversion, a need for cognitive closure, and agreeableness (McCrae et al., 2007; Bonner, 2015; Razavi et al., 2016), which are precursors of employee creativity.

We propose that employees who experience awe in the presence of their supervisors can benefit through enhancement of their self-efficacy by the three components of trust, confidence, and praise (Tierney and Farmer, 2002; Farmer and Tierney, 2017). We argue that supervisor dispositional awe will positively influence employee creative self-efficacy in three areas; social persuasion, task mastery, and physiological well-being (Jiang et al., 2019). A disposition toward positive emotions such as awe has been linked to enhanced persuasion processing (Griskevicius et al., 2010). According to Griskevicius et al. (2010), employees undergoing evaluation rely upon heuristic cues such as a supervisor's expertise and the length of their report for interpreting the quality of feedback they receive from their supervisor (systematic processing). When subordinates feel that their supervisors are acclaimed experts, they regard the feedback they receive as accurate and helpful. If it is positive, it can promote their creative self-efficacy.

Second, supervisor dispositional awe enhances task mastery. Cropley (2006) found that induced awe promoted a higher likelihood of solving tasks through memory recall. Through its two components of perceived vastness and need for accommodation, perceived supervisor dispositional awe will prompt subordinates to apply their existing conventional knowledge in novel ways to solve similar problems in the future (Cropley, 2006). Recalling previous accomplishments boosts an employee's positive affect and creative self-efficacy. Ultimately, dispositional awe can induce higher spiritual intelligence (Bonner, 2015) and an increased grasp of the meaning of life (Moon et al., 2018). It can also promote a spontaneous self-distancing (Le et al., 2019), which positively influences an individual's psychological state and substantially increases feelings of self-efficacy (Moon et al., 2018). In sum, employees experiencing supervisor dispositional awe have higher creative self-efficacy and creativity. Therefore, we posit the following hypothesis:

***Hypothesis 2****: Employee creative self-efficacy mediates the relationship between perceived supervisor dispositional awe and employee creativity*.

### Perceived Supervisor Dispositional Awe as a Moderator

Since the inclusion of creative self-efficacy as an essential correlate of creativity (Tierney and Farmer, 2002; Beghetto and Plucker, 2006), research has explored its mediating effect in China. However, the examination of the mediating role of creative self-efficacy has yielded inconsistent results (Farmer and Tierney, 2017). Li M. et al. (2017) found that creative self-efficacy completely mediated the relationship between proactive personality and innovative work behavior among primary and middle school teachers in China. Zheng and Liu (2017) also used Chinese subjects and found that general self-efficacy was significantly related to creative performance, and this effect was influenced by abusive supervision. Creative self-efficacy partially mediated the relationship between knowledge sharing and employee innovation among 320 supervisor-subordinate dyads in four cities in China (Hu and Zhao, 2016) and between optimism and innovative behavior among Taiwanese undergraduates (Li and Wu, 2011). There was no mediation effect of creative self-efficacy on the relationship between a fixed mindset and a Chinese undergraduate's creative performance (Yunshu et al., 2019). These studies explored creativity-related outcomes such as initiation of independent projects (Boies et al., 2015), the pursuit of challenging tasks (Amabile, 2017), and creative performance (Farmer and Tierney, 2017). There are three potential reasons for these mixed findings: inadequacy in creative self-efficacy measurements, the existence of potential moderators, and the existence of other mediators. Hence, scholars have called for a more in-depth examination of the boundary conditions to determine whether creative self-efficacy mediates the relation between contextual characteristics and creativity by considering the moderating effects of personal traits (Tang et al., 2017). This research attempts to contribute to new information in that area.

Leaders tend to exhibit inconsistent behavior toward their employees: they can be abusive and exude awe simultaneously (Fiset et al., 2019). However, despite the existence of this phenomenon, few studies have explored the interaction between these seemingly opposite leadership characteristics. To address this gap, we drew on the EASI model, which mentions that the expresser's characteristics may change how individuals react to the emotions of others (Van Kleef et al., 2010). We propose that supervisors who inspire awe in susceptible people will tend to share their epiphanies and awe-related experiences with subordinates (Hentrup et al., 2019), which may inspire employees to create new knowledge and ignore negative supervisor behavior. Interaction with awe-inducing charismatic people can cause feelings of self-diminishment (Hentrup et al., 2019). Self-diminishment refers to the perception of being small and insignificant (Piff et al., 2015). This feeling may increase humility in employees (Stellar et al., 2015) and make them more susceptible to persuasion (Piff et al., 2015; Bai et al., 2017) and stimulate them to greater creativity in the workplace (Chirico et al., 2018). Self-diminishment was found to reduce employee's negative reactions to adverse work stressors (Fredrickson, 2004; Li et al., 2019). Supervisor dispositional awe might also provoke subordinates to engage in spontaneous self-distancing when they encounter a problem (Le et al., 2019). According to Le et al. (2019), immersion in awe promotes spontaneous self-distancing in individuals. Spontaneous self-distancing enables subordinates to forego immediate reactance in favor of strategies that might boost their creative self-efficacy (Ayduk and Kross, 2010; Le et al., 2019). Research indicates that awe induced through human interaction motivates individuals to transform from within, and these transformations flow to the whole society (Weber, 1978). Weber's analysis of the same notion illuminates how people such as Steve Jobs, Elon Musk, Barrack Obama, or Nelson Mandela can inspire awe and “reprogram” people to take on heroic and self-sacrificing missions. Therefore, we argue that supervisors with dispositional awe will diminish the negative effect of their abuse on employee creative self-efficacy. In contrast, in situations where there is low supervisor dispositional awe, employees will be overwhelmed with the intensity of abusive supervision, which negatively influences their creative self-efficacy and creative abilities (Oh and Farh, 2017). Summarizing these relationships, we posit that:

***Hypothesis 3:****Perceived supervisor dispositional awe moderates the relationship between perceived abusive supervisory behavior and employee creative self-efficacy, such that the relationship is weaker when supervisor dispositional awe is high rather than low*.

### The Moderated Mediation Model

The present study adopted the EASI model to investigate the direct and indirect moderating effect of supervisor dispositional awe. Previous research has shown that high dispositional awe leads to self-diminishment (Fredrickson, 2004; Li et al., 2019), and spontaneous self-distancing (Ayduk and Kross, 2010; Le et al., 2019) which promotes employee creative self-efficacy and creativity. In contrast, low supervisor dispositional awe is likely to adversely affect employee creative self-efficacy and creativity such that employees will be overwhelmed with the effects of abusive supervisory behavior, become less confident in their creative capabilities, and therefore be less creative in the workplace (Oh and Farh, 2017). Summarizing these relationships, we posit that:

***Hypothesis 4:****Perceived supervisor disposition awe indirectly moderates the relationship between abusive supervisory behavior and employee creativity, such that the effect is weaker when supervisor dispositional awe is high rather than low*.

### Aims and Objectives

The goal of the study was to determine the relationship between perceived abusive supervisory behavior, employee creativity, and the potential mechanisms underlying this association. This study proposed four hypotheses to test this relationship: (1) employee creative self-efficacy mediates the relationship between perceived abusive supervisory behavior and employee creativity; (2) employee creative self-efficacy mediates the relationship between perceived supervisor dispositional awe and employee creativity; (3) perceived supervisor dispositional awe moderates the relationship between abusive supervisory behavior and employee creative self-efficacy; and (4) perceived supervisor disposition awe indirectly moderates the relationship between perceived abusive supervisory behavior and employee creativity, such that the effect is weaker when perceived supervisor dispositional awe is high rather than low.

### Participants

The sample consisted of 223 working professionals undertaking their Master of Business Administration (EMBA) degree at a large university in China. These students who were full-time workers and studied part-time voluntarily took part in the survey.

### Recruitment

Participants were recruited via student network WeChat groups. WeChat was developed by Tencent in 2011 as a social media application, similar to WhatsApp and Telegram. This social media application has evolved into the most extensive stand-alone application in China.

### Sample Criteria and Data Collection

We collected data from working Master of Business Administration (MBA) students who had been on the job for at least 3 months and were pursuing their studies on a part-time basis. We selected this sample because working MBA students are employed in a wide variety of organizations, and this would increase the generalizability of our findings. Research has shown that working MBA students had common factors, such as social class, job level, and relative income, that were relevant to our study outcome. They also met the criteria of being knowledge workers. Davenport (2005, p. 19) stated that “knowledge workers have high degrees of expertise, education, or experience, and the primary purpose of their jobs involves the creation, distribution, or application of knowledge.” We believe that these participants value the creation of knowledge and creative engagement at the workplace for their success. Knowledge workers have also been shown to be more susceptible to abuse from supervisors than their counterparts who have lesser credentials (Tepper, 2007; Tariq et al., 2019).

### Measurements

All scale items were translated from English into Chinese and then back-translated into English to confirm their meaning. All the study variables were measured on 7-point Likert-type scales (1 = strongly disagree; 7 = strongly agree).

#### Perceived Abusive Supervisory Behavior

We used a shortened version of Tepper's (2000) abusive supervision scale to measure perceptions of abusive supervisory behavior. The shortened version has five items that reflect the passive forms of abusive supervisory behavior (ASB) relevant to our study. Sample items included, “My supervisor invades my privacy” and, “My supervisor doesn't give me credit for jobs requiring a lot of effort.” These five items have also been used in similar studies (Mitchell and Ambrose, 2007).

#### Employee Creative Self-Efficacy

We used three items from Farmer and Tierney's (2017) employee creative self-efficacy scale. Representative items included, “I am confident that I could deal efficiently with unexpected events at work” and “I believe that I am good at producing novel ideas.” In this study, creative self-efficacy was characterized by a strong belief in one's general creativity rather than the conviction of being creative in specific fields (Kaufman et al., 2016).

#### Employee Creativity

We measured employee creativity using Scott and Bruce's (1994) six-item creativity scale. Sample items included “I generate creative ideas” and “I promote and champion ideas to others.”

#### Perceived Supervisor Dispositional Awe

We measured supervisor dispositional awe (SDA) using two-items from charismatic attributions scale by Pastor et al. (2002). The sample item included “I would trust my supervisor to overcome any challenge.” We also used three items from a dispositional awe sub-scale (Shiota et al., 2006) that were relevant in the work context. The query items included, “My supervisor seeks out experiences that challenge his/her understanding of the world.” We used a scale related to the attitude of a supervisor toward colleagues rather than workers themselves to avoid desirability and face-related bias. This scale has been used in similar studies (Hentrup et al., 2019).

#### Control Variables

We took into account gender, age, education, and tenure with supervisors, as they been shown to influence employee creativity (Zhou and George, 2001; Shalley and Gilson, 2004). We also controlled for job management, co-worker support for creativity, and personal initiative. Research has demonstrated that co-worker support for creativity made significant independent contributions to creativity (Madjar et al., 2002). To measure job control, participants responded to five items from a scale developed by Jackson et al. (1993). Sample items included, “To what extent are you able to select the methods to apply in your work role?” Co-worker support was measured with three items from Madjar et al.'s (2002) co-worker support scale. Representative queries were: “My co-workers are constantly supportive when I present them with a new concept about my task.” Personal initiative measures the extent to which a person portrays proactive behavior (Frese et al., 1997). A sample item for this scale was “I actively remedy issues at work.”

### Procedures

Before this study, we ensured that the procedures were in line with the ethical standards of the Chinese national research committee and the Helsinki declaration. Consent was obtained from the participants and consisted of the following elements: the purpose of the study, a statement regarding confidentiality, anonymity of participants, and a statement regarding the participant's right to withdraw their consent at any time. The participants also provided their demographic information. An online questionnaire was sent to the participants. The selection criteria were that the participants had been employed at their current workplace for at least 3 months, worked at least 40 h a week (full-time employees), and reported to a supervisor. Twenty-seven subjects did not meet the selection criteria, leaving a sample of 100 and 96 participants. Of these, 69% were female. They had a mean age of 29.80 years (*SD* = 0.66) with an average tenure with the same supervisor of 3.83 years (*SD* = 2.33).

### Data Analysis

To test the proposed hypotheses, we first performed confirmatory factor analyses (CFAs) to establish the discriminant validity of the main study variables. Second, we utilized SPSS version 23.0 to perform the hierarchical multiple regressions to examine the mediating effect of employee creative self-efficacy on the relationship between the proposed independent variables and employee creativity. We then examined the moderating effect of perceived supervisor dispositional awe using hierarchical multiple regression. We first entered the control variables in step 1, the independent variable in step 2, and the moderator in step 3. We mean-centered all the component variables required to create the interaction term, perceived abusive supervisory behavior (ASB), and perceived supervisor dispositional awe (SDA). We then entered the interaction term (ASB × SDA) in step 4. Finally, we carried out a bias-corrected bootstrapping procedure using PROCESS macro, Model 8, to test the moderated mediated model (Hayes, 2013).

### Preliminary Analyses

As all variables in our study were collected from a single source, we needed to check the common method bias of our data. To address this concern, we followed several recommendations during the research design and analysis phases (Podsakoff et al., 2003, 2012). In the research design phase, we assured participants of the anonymity of the survey and the confidentiality of the data. We also simplified some statements to increase respondents' understanding of the questions. Finally, during the analysis phase, a series of confirmatory factor analyses were carried out. Harman's one-factor test was conducted with an unrotated factor solution. The test revealed an explained variance of 31.56%, which is below the threshold of 50% suggested by Podsakoff et al. (2003). Harman's single factor was also run using CFA. Researchers have shown that method biases were substantial when a single factor model fits the data (Serrano-Archimi et al., 2018), so we performed a CFA to ensure that all our scales were empirically distinct. The four-factor model showed the best-fit indices, $\chi_{\left(146\right)}^{2}$ = 553.29, comparative fit index, CFI = 0.90, and the root mean square error of approximation, RMSEA = 0.11. It also provided a significantly better fit to the data than (a) a three-factor model, where perceived ASB and perceived SDA combined into one factor [$\chi_{\left(149\right)}^{2}$ = 1098.76; CFI = 0.73; RMSEA = 0.18], (b) a three-factor model, where perceived SDA and employee creative self-efficacy were combined into one factor [$\chi_{\left(149\right)}^{2}$ = 924.61; CFI = 0.78; RMSEA = 0.16], (c) a three-factor model where perceived ASB and employee creative self-efficacy were combined into one factor [$\chi_{\left(149\right)}^{2}$ = 915.49; CFI = 0.79; RMSEA = 0.16], and (d) a single factor [$\chi_{\left(152\right)}^{2}$ = 2458.60; CFI = 0.36; RMSEA = 0.28]. These results are outlined in Table 1 and confirm that CMV is not a major issue in our data (Gaski, 2017).

**Table 1:** The results of confirmatory factor analysis.

		**CR**	**AVE**	**MSV**	**MaxR (H)**	**1**	**2**	**3**	**4**
1	Creative self-efficacy	0.88	0.72	0.09	0.91	0.84			
2	Perceived SDA	0.92	0.72	0.17	0.96	0.17^*^	0.85		
3	Perceived ASB	0.95	0.82	0.08	0.96	−0.28^***^	−0.21^**^	0.90	
4	Employee creativity	0.97	0.86	0.17	0.98				0.93

*N = 196*.

Significant at:

* p < 0.05;

** p < 0.01; and ***p < 0.001.

*SDA, supervisor dispositional awe; ABS, abusive supervisory behavior; CR, composite reliability; AVE, average variance extracted; MSV, maximum shared variance; MaXR (H), maximum reliability*.

## Results

**Figure 2:**
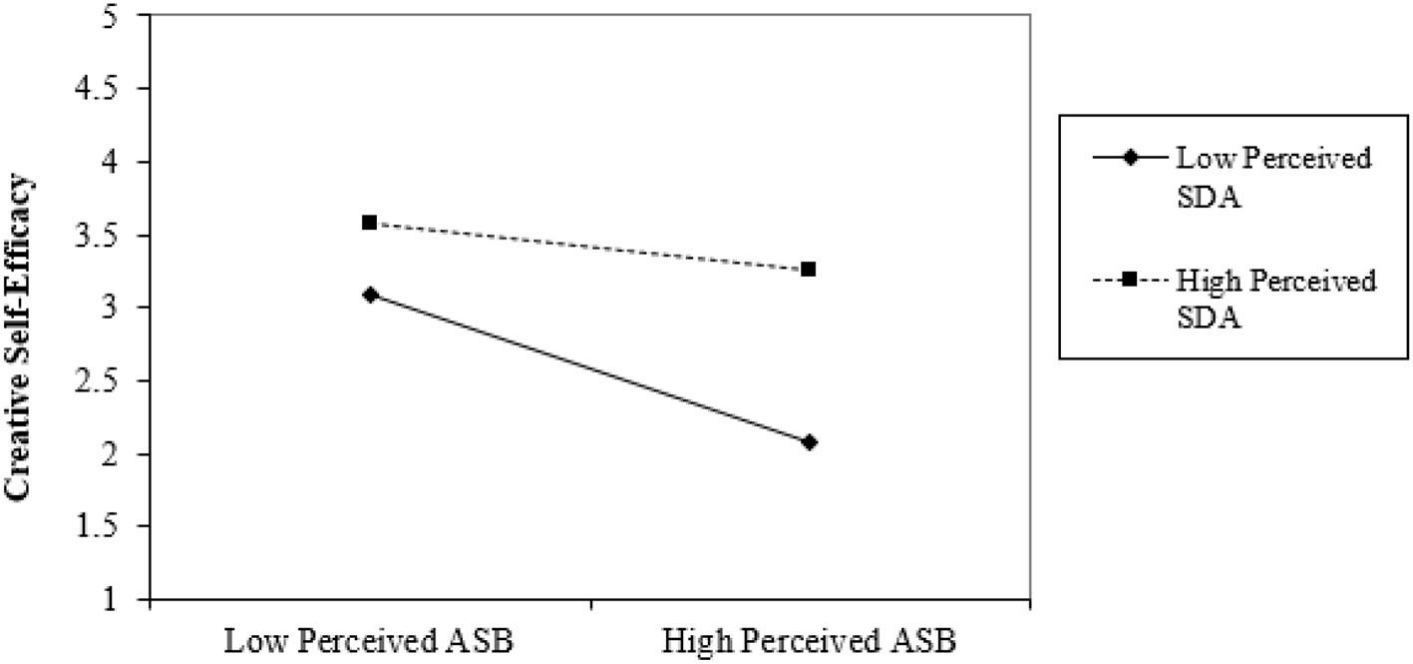
The moderating effect of perceived SDA (perceived supervisor dispositional awe) on the relationship between perceived ASB (perceived abusive supervisor behavior) and employee creative self-efficacy.

The mean, standard deviation, and correlation for all variables are summarized in Table 2. Perceived ASB was observed to be negatively related to employee creative self-efficacy (β = –0.26, *p* < 0.01) and creativity (β = –0.26, *p* < 0.05). Perceived SDA was positively related to employee creative self-efficacy (β = 0.14, *p* < 0.05), and creativity (β = 0.16, *p* < 0.05). Employee creative self-efficacy was positively associated with employee creativity (β = 0.85, *p* < 0.01). Also, creative self-efficacy fully mediated the relationship between perceived ASB and employee creativity (β = 0.34*, p* < 0.01; Table 3, Model 6) and partially mediated the relationship between perceived SDA and employee creative self-efficacy (β = 0.87*, p* < 0.01; Table 4, Model 6). Table 5 shows that the interaction between perceived ASB and perceived SDA was positively correlated with employee creative self-efficacy (β = 0.10*, p* < 0.05). Table 6 shows that the indirect effect of perceived SDA on employee creativity via employee creative self-efficacy at work was significant for both low and high levels of perceived SDA. These results support hypotheses 1–4. The interaction plot in Figure 2 indicates that experiencing awe allows the employee to maintain creative self-efficacy despite being abused.

**Table 2:** Mean, standard deviations, correlation, and square roots of AVE in diagonals.

		***M***	***SD***	**1**	**2**	**3**	**4**	**5**	**6**	**7**	**8**	**9**	**10**	**11**
1	Gender	1.59	0.49	1										
2	Age	1.24	0.66	−0.20^**^	1									
3	Education	3.21	1.93	−0.18^*^	−0.06	1								
4	Tenure with supervisor	2.86	2.33	−0.22^**^	0.50^**^	0.50^**^	1							
5	Co-worker support	4.98	1.04	−0.11	−0.09	0.08	−0.05	(0.87)						
6	Job control	5.28	1.37	−0.04	−0.07	0.05	0.11	−0.05	(0.92)					
7	Personal initiative	5.17	0.84	−0.11	−0.02	0.01	−0.01	0.72^**^	−0.15^*^	(0.81)				
8	Perceived ASB	4.79	1.60	−0.09	−0.03	−0.09	−0.14	0.19^**^	−0.192^**^	0.24^**^	(0.95)			
9	Perceived SDA	4.73	1.44	0.10	0.06	−0.02	0.07	0.02	−0.13	0.13	−0.16^*^	(0.92)		
10	Employee creative self-efficacy	4.95	1.60	0.02	0.05	−0.01	0.05	−0.10	0.13	0.06	−0.26^**^	0.14^*^	(0.88)	
11	Employee creativity	4.84	1.66	0.05	0.03	−0.08	0.00	−0.05	0.12	0.07	−0.26^**^	0.16^*^	0.85^**^	(0.97)

*N = 196*.

*ASB, abusive supervisory behavior; SDA, supervisor dispositional awe. Values in parentheses on the diagonals are the square roots of AVE of each scale*.

* p < 0.05 and **p < 0.01.

**Table 3:** Employee creative self-efficacy as mediator of perceived abusive supervisory behavior and employee creativity.

	**Creative self-efficacy**				**Employee creativity**							
			**95% C.I**.									
	**Model 1**	**Model 2**	**LL C.I**.	**UL C. I**.	**Model 3**	**Model 4**	**LL C.I**.	**UL C. I**.	**Model 5**	**Model 6**	**LL C.I**.	**UL C.I**.
Age	0.14 (0.22)	0.14 (0.22)	−0.28	0.57	0.08 (0.23)	0.09 (0.22)	−0.35	0.53	−0.11 (0.18)	−0.15 (0.16)	−0.49	0.21
Gender	0.12 (0.23)	0.12 (0.23)	−0.42	0.49	0.20 (0.25)	0.10 (0.24)	−0.37	0.58	−0.14 (0.19)	−0.19 (0.18)	−0.55	0.33
Education	0.02 (0.07)	0.02 (0.07)	−0.13	0.15	−0.06 (0.08)	−0.07 (0.07)	−0.22	0.08	−0.05 (0.06)	−0.05 (0.05)	−0.17	0.00
Tenure	−0.01 (0.07)	−0.01 (0.07)	−0.17	0.10	0.00 (0.07)	−0.01 (0.07)	−0.16	0.13	0.00 (0.06)	0.00 (0.05)	−0.10	0.09
Co-worker support	−0.49 (0.16)	−0.49 (0.16)^**^	−0.78	−0.17	−0.35 (0.16)^*^	−0.33 (0.16)^*^	−0.65	−0.01	−0.14 (0.13)	0.03 (0.12)	−0.21	0.27
Job control	0.20 (0.08)	0.20 (0.08)^**^	−0.01	0.31	0.19 (0.09)^*^	0.14 (0.08)	−0.02	0.31	0.10 (0.07)	0.02 (0.06)	−0.10	0.10
Personal initiative	0.62 (0.20)	0.62 (0.20)^**^	0.33	1.10	0.52 (0.21)^*^	0.63 (0.20)^*^	0.22	1.03	0.71 (0.16)^**^	0.51 (0.15)^*^	0.20	0.22
Perceive ASB		0.14 (0.22)	−0.42	−0.13	0.08 (0.23)	−0.30 (0.07)^**^	−0.45	−0.15		−0.03 (0.05)	−0.15	0.02
Creative self-efficacy										0.34 (0.05)^**^	0.23	0.95
*R*^2^	07^*^	0.14^**^			0.06	0.13^**^			0.06	0.73^**^		
Δ*R*^2^	0.07^*^	0.06^**^			0.06	0.07^**^			0.06	0.67^**^		

*N = 196*.

*ASB, abusive supervisory behavior*.

* p < 0.05 and **p < 0.01.

**Table 4:** Employee creative self-efficacy as mediator of perceived supervisor dispositional awe and employee creativity.

**Employee creative self-efficacy**								**Employee creativity**				
			**95% C.I**.								**95% C.I**.	
	**Model 1**	**Model 2**	**LL C.I**.	**UL C. I**.	**Model 3**	**Model 4**	**LL C.I**.	**UL C. I**.	**Model 5**	**Model 6**	**LL C.I**.	**UL C.I**.
Age	0.14 (0.22)	0.13 (0.22)	−0.30	0.57	0.08 (0.23)	0.08 (0.23)	−0.38	0.54	0.08 (0.23)	−0.03 (0.12)	−0.28	0.20
Gender	0.12 (0.23)	0.07 (0.24)	−0.40	0.54	0.20 (0.25)	0.13 (0.25)	−0.36	0.62	0.20 (0.25)	0.07 (0.13)	−0.19	0.33
Education	0.02 (0.07)	0.02 (0.07)	−0.12	0.17	−0.06 (0.08)	−0.05 (0.07)	−0.21	0.10	−0.06 (0.08)	−0.08 (0.04)^*^	−0.16	0.00
Tenure	−0.01 (0.07)	−0.02 (0.07)	−0.16	0.11	0.00 (0.07)	−0.00 (0.07)	−0.15	0.14	0.00 (0.07)	0.01 (0.04)	−0.06	0.09
Co-worker support	−0.49 (0.16)^*^	−0.47 (0.16)^*^	−0.79	−0.15	−0.35 (0.16)^*^	−0.32 (0.16)^*^	−0.65	0.00	−0.35 (0.16)^*^	0.08 (0.09)	−0.09	0.28
Job control	0.20 (0.08)^*^	0.21 (0.08)^*^	0.05	0.38	0.19 (0.09)^*^	0.21 (0.08)^**^	0.04	0.39	0.19 (0.09)^*^	0.00 (0.04)	−0.08	0.11
Personal initiative	0.62 (0.20)^*^	0.57 (0.20)^*^	0.17	0.96	0.52 (0.21)^*^	0.45 (0.21)^*^	0.04	0.87	0.52 (0.21)	−0.00 (0.11)	−0.23	0.18
Perceived SDA		0.14 (0.08)	−0.01	0.30		0.17 (0.08)^*^	0.01	0.34		−0.06 (0.04)	−0.14	0.14
Creative self-efficacy										0.87 (0.04)^**^	0.79	0.96
*R*^2^	0.07^*^	0.14^**^			0.06^*^	0.08^*^			0.06	0.73^**^		
Δ*R*^2^	0.07^*^	0.06^**^			0.06^*^	0.02^*^			0.06	0.67^**^		

*N = 196*.

*SDA, supervisor dispositional awe*.

* p < 0.05 and **p < 0.01.

**Table 5:** Results of the moderating effect of perceived supervisor dispositional awe.

	**Employee creative self-efficacy**				
	**Model 1**	**Model 2**	**Model 3**	**95% C.I**.	
				**LL C.I**.	**UL C.I**.
Age	−0.11 (0.18)	−0.10 (0.18)	−0.17 (0.17)	−0.52	0.17
Gender	−0.14 (0.19)	−0.23 (0.19)	−0.24 (0.19)	−0.62	0.12
Education	−0.05(0.06)	−0.05 (0.06)	−0.08 (0.06)	−0.20	0.03
Tenure	0.00 (0.06)	−0.02 (0.05)	−0.00 (0.05)	−0.11	0.11
Co-worker Support	−0.14 (0.13)	−0.07 (0.13)	−0.07 (0.13)	−0.33	0.18
Job Control	0.10 (0.07)	0.08 (0.07)	0.05 (0.06)	−0.07	0.19
Personal Initiative	0.71 (0.16)^**^	0.60 (0.17)^*^	0.60 (0.17)^*^	0.26	0.94
Perceived ASB		−0.15 (0.06)^*^	−0.17 (0.05)^*^	−0.29	−0.05
Perceived SDA		0.20 (0.09)^*^	0.25 (0.08)^*^	0.07	0.42
ASB × SDA			0.14 (0.04)^*^	0.06	0.23
*R*^2^	0.14^**^	0.18^*^	0.23^*^		
Δ*R*^2^	0.14^**^	0.04^*^	0.04^*^		

*N = 196*.

*ASB, abusive supervisory behavior; SDA, supervisor dispositional awe*.

* *p < 0.05*,

** p < 0.01, and ***p < 0.001.

**Table 6:** Results of the moderated path analysis.

**Moderator**	**Perceived ASB (X)** ** → employee creative self-efficacy (M)** ** → employee creativity (Y)**		
	**Indirect effects**	**Boot SE**	**95% C.I**
Low levels of perceived SDA (−1 *SD*)	−1.53	0.04	[−0.256, −0.077]
Mean levels of perceived SDA	−0.96	0.02	[−0.162, −0.048]
High levels of perceived SDA (+1 *SD*)	−0.04	0.02	[−0.096, 0.000]

*n = 196. Bootstrap N = 10,000*.

*ASB, perceived abusive supervisory behavior; SDA, supervisor dispositional awe; P_MX_, path from abusive supervisor behavior to employee creative self-efficacy; P_YM_, path from employee creative self-efficacy to employee creativity; P_YX_, path from abusive supervisor behavior to employee creativity*.

*p < 0.05 and **p < 0.01.

## Discussion

The current study builds upon prior work (Fiset et al., 2019; Hentrup et al., 2019) to examine the interaction between perceived abusive supervisory behavior and perceived supervisor dispositional awe on follower creative self-efficacy and creative performance. Our results revealed that perceived abusive supervisory behavior negatively predicted employee creativity, and creative self-efficacy fully mediated this relationship. Also, perceived supervisor dispositional awe positively predicted employee creativity; and employee creative self-efficacy partially mediated this relationship. These findings are in line with previous research on the mediating effects of creative self-efficacy (Hu and Zhao, 2016; Zheng and Liu, 2017). Previous research has proven that creative self-efficacy is influenced by leadership and predicts employee creativity (Zheng and Liu, 2017). The influence of leadership can be explained by Rank et al.'s (2004) assertion that employees, after experiencing or observing abusive behavior, intentionally diverted their efforts to other performance dimensions where their self-efficacy was assured. To explain the partial mediating effect of employee creative self-efficacy, we turn to Hu and Zhao's (2016) argument that although creative self-efficacy is a stable and consistent predictor of employee creativity, it must be aligned with environmental factors to be effective.

In line with the EASI model (Van Kleef et al., 2009; Van Kleef, 2016, 2017), we found the relationship between perceived abusive supervisory behavior, employee creative self-efficacy, and creative performance depended on the strength of their disposition to perceive the leader as awe-inspiring. In particular, the results suggest that a high degree of perceived dispositional awe of a supervisor buffers the effects of ASB on employee creativity. This finding implies that perceived abusive supervisor behavior and perceived supervisor dispositional awe interact to play an essential role in how followers evaluate their level of creative self-efficacy, which guides their creative performance. As leader profiles indicate the ability for leaders to simultaneously demonstrate positive and negative leadership characteristics (Isaacson, 2012; Fiset et al., 2019; Hentrup et al., 2019), these findings make an essential contribution to the literature, enabling us to understand better how such interactions influence creative performance.

### Theoretical Implications

This study made two notable theoretical contributions. First, the study addressed the problem of inconsistent findings related to the link between perceived abusive supervisory behavior, perceived supervisor dispositional awe, and employee creativity by incorporating the EASI model. We demonstrated that employees considered perceived abusive supervisory behavior as a negative emotional expression and perceived supervisor dispositional awe as a positive emotional expression that influenced employee cognition and behavior. We contributed to the EASI model (Van Kleef, 2009, 2016, 2017) by examining employee inferential responses to their supervisor's emotional expressions such as creative self-efficacy. Also, by focusing on creative self-efficacy as a mediator, the study provided a moderator that explained the inconsistencies in the literature (Yunshu et al., 2019). To our knowledge, this is the first study to explore the mediating effect of employee creative self-efficacy on the relationship between perceived supervisor dispositional awe and employee creativity. Later it examined the direct and indirect moderating effect of perceived supervisor dispositional awe on the relationship between perceived abusive supervisory behavior, employee creative self-efficacy, and employee creativity. The study incorporated perceived negative and positive supervisor characteristics to contribute to the literature on abusive supervision (Namie and Namie, 2000; Tepper et al., 2011; Vogel and Mitchell, 2017) and charismatic leadership (Pastor et al., 2002; Hentrup et al., 2019). Previous research had ignored a group of leaders who exhibited abusive behavior but were celebrated in corporate circles for their creative paradigm shifts (Oh and Farh, 2017).

Second, the study contributed to the literature of positive psychology by examining the buffering effect of awe (Gordon et al., 2017; Hentrup et al., 2019) on work-related stressors. Our work expanded the scope of analysis from the general therapeutic benefits of awe (Fredrickson and Joiner, 2002; Stellar et al., 2015) to a specific work-related stressor such as perceived abusive supervisory behavior (Fredrickson and Joiner, 2002; Stellar et al., 2015). A disposition toward awe of supervisors provided employees with an ability to spontaneously self-distance (Le et al., 2019) and self-diminish (Piff et al., 2015), which indirectly contributed to workplace creativity (Chirico et al., 2018). It seems that subordinate perceptions of abusive supervisory behavior were prone to fluctuate because of the inspirational impact they had on their subordinates.

### Practical Implications

Our results provide additional evidence that abusive supervisory behavior is harmful to employees' creativity as it decreases their creative self-efficacy, but that the leader's ability to invoke awe can buffer this aversive tendency. As leaders who are engaged in abusive behavior are often unaware of the harm their actions cause, they may exhibit awe-inducing behavior to mitigate the effect of their unconscious detrimental deeds. In other words, supervisors may intentionally inspire followers by attracting their attention to awe-related experiences, as this may help to protect against the adverse effects of abusive supervision. In sum, organizational leaders can try to minimize abusive behavior, or at least they may train supervisors to induce dispositional awe to balance its adverse effects (Mackey et al., 2017).

### Limitations and Future Directions

The current study has several limitations that provide avenues for future research. First, as with any cross-sectional study, we cannot make causal inferences based on the results of our research. For example, mistreatment literature (Vogel and Mitchell, 2017) suggests that low dispositional tendencies of observers or victims may potentially influence perceptions of abuse (Tepper et al., 2011). Research has shown that subordinates that rate highly in trait neuroticism and negative affectivity have biased perceptions toward abusive supervision (Oh and Farh, 2017). These dispositions correspond to the dimension of creativity. Thus, future research may adopt a longitudinal or laboratory experiment design to establish a clear connection between abusive supervisory behavior and creative self-efficacy while considering personality traits (Vogel and Mitchell, 2017).

Second, since the results are based on self-reported measures, they might be inflated due to common method bias. However, we adhered to the guidelines while performing the discriminant validity and confirmatory factor analyses, and the literature has indicated that self-reported measures of creativity were just as informative as objective measures (Zhou and George, 2001).

Third, the variables in the current study were all collected from the same source, the employees. While common-source bias may arise, our testing of the moderation and moderated mediation hypotheses showed that it did not affect the results. Future experimental or longitudinal research is needed to confirm the proposed causal relationships of the variables by manipulating perceived awe and creativity as well as creative self-efficacy.

Fourth, research has shown that the magnitude and content of different constructs vary in different socio-cultural contexts (Bai et al., 2017). The current study demonstrated variations of creative self-efficacy, abusive supervision, supervisor dispositional awe, and creativity in cross-cultural contexts among Chinese workers. The Chinese population, in general, has been known to have high power distance orientation and to follow a collectivist culture (Farh et al., 2007). The results suggest that the relationship between perceived abusive supervisory behavior and employee creative self-efficacy will vary in other cultures, such as those with greater emphasis on individual rights or that are lower in power distance orientation. We leave these assumptions for future investigators to test.

Previous research has indicated that low appraisals are event-specific and may vary when the abuse is continuous (Tepper et al., 2011; Oh and Farh, 2017). Continuous abuse may induce passivity in subordinates, which may negatively influence their creative performance despite environmental and situational changes. Thus the buffering effect of supervisor dispositional awe may play only a short-term, tactical role in the face of poor performance, as opposed to long-term adverse interpersonal treatment perpetuated by abusive supervisors (Tepper et al., 2011). Therefore, future research should compare the effects of the supervisor's dispositional awe on abuse that is repeated or continuous. Knowledge in this area might benefit from investigating the impact of perceived abusive supervisory behavior on team creativity (Priesemuth and Schminke, 2019). Future work should strive to understand how abusive supervisory behavior affects group-level cognition and emotional reactions and how these states influence teams' desires to be creative and the process of creativity itself. We anticipate that group-level analyses will follow a trend similar to what was observed in the current study. Future work should empirically test this assertion as the focus of our research was limited to creativity as an outcome of perceived abusive supervision. Future research might explore the interactive effect of abusive supervisory behavior and supervisor dispositional awe on other organizational variables, such as employee job satisfaction, job engagement, and citizenship behavior on the individual and group level.

## Conclusions

Grounded in the emotions as based on social information (EASI) model, the current study explored how perceived abusive supervisors influenced subordinates' creativity. We surveyed employees to understand better the moderating effect of supervisor dispositional awe on the relationship between perceived abusive supervisor behavior and creativity via employee creative self-efficacy. The current study answered a call to explore examples of abusive supervisor behavior that were less detrimental, and our findings support the idea that supervisors who induce dispositional awe in their subordinates may be able to mitigate the effects of abuse on creative performance. These insights can inform our understanding of the impact of inconsistent leadership behavior on employee cognition and actions. We hope to motivate future researchers to explore other leadership traits of supervisors and their different effects on organizational outcomes.

## Data Availability Statement

The datasets generated for this study are available on request to the corresponding author.

## Ethics Statement

The studies involving human participants were reviewed and approved by Chinese Academy of Sciences Research Ethics Committee. The patients/participants provided their written informed consent to participate in this study.

## Author Contributions

CA conceived of and wrote the manuscript. AP and FI contributed writing, figures, and edits. All authors contributed to the article and approved the submitted version.

## Conflict of Interest

The authors declare that the research was conducted in the absence of any commercial or financial relationships that could be construed as a potential conflict of interest.
